# Understanding long-term opioid prescribing for non-cancer pain in primary care: a qualitative study

**DOI:** 10.1186/s12875-015-0335-5

**Published:** 2015-09-11

**Authors:** Carolyn McCrorie, S. José Closs, Allan House, Duncan Petty, Lucy Ziegler, Liz Glidewell, Robert West, Robbie Foy

**Affiliations:** Leeds Institute of Health Sciences, Charles Thackrah Building, University of Leeds, 101 Clarendon Road, Leeds, LS2 9LJ UK; School of Healthcare, Baines Wing, University of Leeds, Leeds, LS2 9JT UK; Bradford School of Pharmacy, University of Bradford, Richmond Rd, Bradford, Yorkshire BD7 1DP UK

## Abstract

**Background:**

The place of opioids in the management of chronic, non-cancer pain is limited. Even so their use is escalating, leading to concerns that patients are prescribed strong opioids inappropriately and alternatives to medication are under-used. We aimed to understand the processes which bring about and perpetuate long-term prescribing of opioids for chronic, non-cancer pain.

**Methods:**

We held semi-structured interviews with patients and focus groups with general practitioners (GPs). Participants included 23 patients currently prescribed long-term opioids and 15 GPs from Leeds and Bradford, United Kingdom (UK). We used a grounded approach to the analysis of transcripts.

**Results:**

Patients are driven by the needs for pain relief, explanation, and improvement or maintenance of quality of life. GPs’ responses are shaped by how UK general practice is organised, available therapeutic choices and their expertise in managing chronic pain, especially when facing diagnostic uncertainty or when their own approach is at odds with the patient’s wishes. Four features of the resulting transaction between patients and doctors influence prescribing: lack of clarity of strategy, including the risk of any plans being subverted by urgent demands; lack of certainty about locus of control in decision-making, especially in relation to prescribing; continuity in the doctor-patient relationship; and mutuality and trust.

**Conclusions:**

Problematic prescribing occurs when patients experience repeated consultations that do not meet their needs and GPs feel unable to negotiate alternative approaches to treatment. Therapeutic short-termism is perpetuated by inconsistent clinical encounters and the absence of mutually-agreed formulations of underlying problems and plans of action. Apart from commissioning improved access to appropriate specialist services, general practices should also consider how they manage problematic opioid prescribing and be prepared to set boundaries with patients.

## Background

Opioids are commonly prescribed for chronic, non-cancer pain despite limited evidence of effectiveness and frequent adverse effects [1–4]. There are growing concerns that strong opioids are prescribed inappropriately and alternative approaches under-utilised [5–9]. Community prescribing of opioids in England has more than doubled in the last decade, with larger increases for more potent opioids [10, 11] reflecting international trends [12–15]. Over a third of patients eventually more than double their original dose of opioids and around 10 % are prescribed potentially hazardous high doses [12]. Rather than reflecting previously untreated chronic pain, these patterns may reflect a wider change in the general approach to prescribing for pain [5, 16].

The therapeutic benefit of opioids in musculoskeletal pain is established for short but not long term use [8, 10, 12] whilst evidence is accumulating on harms, most commonly physical dependence, tolerance, addiction and adverse effects [8, 17–21]. Many people with chronic pain report difficulties with prescribed opioids, including psychosocial problems and concerns about controlling their medication use [22]. Non-adherence to treatment plans is common because of side effects or fears about dependence [23]. Many patients report poorly controlled pain, often resorting to self-medication and diversion as coping strategies [22, 24–27]. Hospitalisation rates due to opioid-related adverse events are increasing [28]. Opioid use, even of the commonly prescribed and ‘weaker’ codeine, is associated with increased mortality [29].

Patients with chronic pain face challenges, such as explaining and legitimising their symptoms [30], whilst general practitioners (GPs) report difficulties in assessing pain and concerns about prescribing strong opioids [21, 31–35]. Patients and GPs often find the management of chronic pain unsatisfactory.

We interviewed patients and GPs about their experiences, beliefs and expectations of analgesia prescribing, to improve understanding of how problematic long-term opioid prescribing becomes established.

## Methods

### Design and setting

A qualitative study, comprising semi-structured interviews and focus groups, in general practices across Leeds and Bradford, cities in the North of England.

### Sampling

Thirty-seven (23 %) of 158 general practices approached expressed an interest. To ensure diversity in their experiences, we sampled patients from practices with high and low opioid prescribing levels. Electronic searches identified patients aged 18 and over, with a current repeat prescription for either a strong opioid (e.g. morphine) or a weak opioid (e.g. codeine). We excluded patients with cancer or cognitive impairment. Each practice invited up to 60 patients by letter to contact the study team. As recruitment progressed, we purposively sampled by sex, age, recent significant changes in opioid prescriptions, opioid strength, the presence of coded mental health problems and ethnicity in order to maximise the range of relevant attributes.

We invited individual GPs from the 37 practices to take part in a focus group held in their locality, seeking one GP from each practice.

### Data collection

Semi-structured interviews (by CM and LZ) with individual patients covered accounts of their prescribing histories, experiences of treatment, and hopes and expectations (Appendices 1 and 2).

Early patient interviews informed the development of patient vignettes to prompt the subsequent focus group discussions with GPs (facilitated by RF and CM). The two vignettes illustrated patients experiencing suboptimal pain control on opioids. We prompted discussion on experiences and expectations of the initiation and maintenance of opioid prescribing, management options, perceived ‘red flags’ for long-term opioid use and experiences of trying to reduce prescribing. All interviews and focus groups were audio taped and transcribed verbatim following written consent.

### Analysis

Constant comparison was used to analyse transcriptions. Analysis involved deconstructing each transcript to identify primary categories (open coding). These categories were compared with others within and across transcripts, followed by cross-linking of categories to generate new meanings and concepts (axial coding) then cross-linking of concepts to generate themes (selective coding) [36]. Interviews and focus groups were initially coded separately. Coding reliability was checked by two further researchers versed in qualitative analysis (SJC and LZ).

Data collection and analysis proceeded iteratively, with focus group findings informing later patient interviews, allowing progressive focusing on key themes to explain findings. The wider study team also read selected transcripts before discussing emerging findings and analyses. From the two perspectives (patient and GP) we identified, we derived an account of the main features of transactions between patients and GPs and subsequent prescribing decisions.

### Ethical approval

The study was approved by Yorkshire and the Humber (Humber Bridge) NRES Committee 12/YH/0109.

## Results

Sixty-one out of 391 invited patients expressed an interest. We subsequently interviewed 23 patients (17 women; mean age 54 years; range 31 to 89 years; Table 1). Fifteen patients were on stable, prescribed, mostly strong, opioids. Five had stepped up opioid strength within the preceding year and three had stepped down. The mean duration of opioid use was 14 years (range two to 35 years). There were discrepancies between prescribing records and patient self-reports. For example, three patients indicated that they were no longer taking prescribed opioids; one because of recommendations from a specialist and two because of personal choice. All had experienced adverse side effects coupled with minimal or no pain relief. We interviewed 21 patients at their homes, one at a general practice and one at the University. Spouses were present in two interviews. We held 12 interviews before the first GP focus group and six were held following the second GP focus group. Mean interview duration was 53 minutes (range 34 to 77 minutes).

**Table 1:** Patient sample characteristics

Area of residence	Bradford	12
	Leeds	11
Female		17
Median age in years (range)		54 (31–89)
Mean Index of Multiple Deprivation (SD)		20 (12)
Coded mental illness		17
Current opioid strength	Strong	15
	Weak	5
	Non-opioid	3
Trajectory (changes within the preceding year)	Step-up	5
	Step-down	3
	Stable	15
Total		23

Fifteen GPs (11 female) from 13 practices (five urban and eight rural) participated in two focus groups. One GP had agreed to participate but subsequently did not attend. Participants had on average 10 years’ experience as GPs (range two to 36 years) and a median list size of 7500 patients (2400 to 25000 patients). Two GPs had special interests in pain management, one in mental health and one in palliative care.

### Patient drivers to seek help

Patients sought pain relief, an explanation of their symptoms and to improve or maintain quality of life. Such hopes and expectations often continued to be unmet amongst those patients we interviewed. Sometimes this was because expectations of achievable pain relief were unrealistic:

*‘[I wanted the GP] just to relieve the pain really… more or less just give me the medication to take the pain away. Nothing other than that.’* [Male, 61 yrs, maintained on weak opioids, D1; 64&208]

Patients repeatedly consulted with poorly controlled pain despite continuing or even increased doses and strengths of pain killers. However, they often recognised that they had to balance any benefits from pain relief with the adverse effects of opioids. Hopes for pain relief were tempered by fears about adverse effects of treatment escalation, particularly opioid dependence:

*‘… and from what I’ve read up, because I like to, sort of, keep on top of things, that it’s an opium based drug, so you will build up some tolerance and you will build up* [becomes tearful] *And you will potentially become sort of addicted to it, if you like.’* [Male, 47 yrs, increasing strong opioids, C2; 62]

Patients sought an explanation for their symptoms and often struggled to receive one. The patients we interviewed often described complex narratives, sometimes relating the onset of their symptoms to specific events (such as accidental injuries) and often requiring multiple investigations and consultations with primary and secondary care clinicians. They picked up partial or even contradictory explanations for their symptoms; diagnostic labels such as osteoarthritis, fibromyalgia and hyper-mobility syndrome did not appear to sufficiently capture their suffering. Doctors’ explanations which attributed poorly controlled pain symptoms to lifestyle factors or life events were received with or even fuelled scepticism:

*‘Well it was like a challenge for me…because all they did really was to tell me to lose weight, it’s the menopause, and that was it…and I kept going to the GP because I knew something wasn’t right. But I had the stamina to say well I know something’s not right, I want to be seen.’* [Female, 54 yrs, maintained on strong opioids, G4; 38–54]

Patients’ abilities to cope with pain varied over time, and they were driven to seek help when medication or other coping measures no longer work and their pain interferes with their usual activities. The centrality of quality of life to patients was apparent in their distress when seeking help from GPs:

*‘I don’t know how many times I’ve sat and I’ve just sat and cried and said, I’ve had enough, you know. And before I got on the gabapentin I just sat in the GP’s office in floods of tears like a lumbering idiot and said if you can’t take this pain away from my legs just chop them off, because it’s just 24/7…’* [Female, 37 yrs, maintained on weak opioids, G2; 105]

### Features of transactions between patients and GPs

Four themes characterised not just the views of patients or GPs, but some feature of the transactions between them.

*Absence of a shared management plan.* First and most importantly, the management of patients with chronic pain often unfolded in the absence of a shared long-term management plan so that prescribing occurred as a default reaction. Prescribing decisions for unfamiliar patients, often with long and complex pain histories, were made within the time constraints of routine consultations:

*‘They’ll be down for their medication review but they’ll say, I’m here for my medication review, you’ll spend some time doing the tablets. Oh and whilst I’m here I’ve got this rash, I’ve got this, whatever it is. So that automatically then reduces the time you can actually spend looking at all of their medication which might include their painkillers.’* [GP, female, Leeds focus group; 246]

Pain management was often marginalised in the context of co-morbidities, especially given the absence of clear treatment goals compared with other long-term conditions, such as diabetes.

Patients could also receive opioid prescriptions after telephone requests, when GPs were juggling various other urgent calls and queries competing for their time. GPs resorted to the compromise of prescribing further opioids without formal medication reviews. These short-term fixes accumulated so that repeated prescriptions or escalating strengths of opioid became the norm for patients until either party realised that the clinical management approach was not working. At this point, safety concerns may have been raised or the GP may have admitted to running out of ideas on further management:

*‘It seems to me that when I got to a certain level [300mcg Fentanyl patch], it was almost like she’d [the GP] not realised that she’d gotten me up to that....and so now all we do is work on getting that level down.’* [Female, 37 yrs, reducing strong opioids, B3; 162, 174]

GPs were aware of the limitations of prescribed opioids, which then compromised their abilities to formulate coherent management plans. They expressed some uncertainty about how to prescribe them appropriately and were aware of giving conflicting advice to patients on balancing pain relief against the risks of tolerance and addiction:

*‘… there’s something about us not saying to patients often enough, you know, these are addictive, take them when you’re in pain. Because everybody always says, you need to take them four times a day because if you break that… your pain is going to come through. So we’re telling them that on the one side and then on the other side we’re saying, you’ve got to stop taking it as soon as you’re not in any pain because otherwise you’re going to be hooked on them and your use is going to escalate up.’* [GP, female, Bradford; 736]

*Locating control and responsibility for change.* Second, we recognised a discussion about where perceived control resided and who has the ability to make change. Sometimes patients positioned control with their GPs:

*‘And I know the doctors know what they are doing, I suppose, but you don’t know whether to say ‘I think these patches are working, can you up it?’* [Female, 72 yrs, maintained on strong opioids, A1;126]

At other times, patients felt responsible for controlling the use of prescribed medication. Not infrequently, neither party felt in control and non-pharmacological approaches were limited by resource constraints:

*‘He [the GP] said what exactly do you want me to do? You've pretty much been on everything so there is nothing for you to do. There is nothing for you to go on to. And I was like well what exactly am I doing coming off [strong opioids] when you've got nothing to put me on to?'* [Female, 37 yrs, reducing strong opioids, B3; 140]

One response to this mutual lack of control was to refer patients on for specialist assessment and management, usually to pain clinics. Access to appropriate pain services, especially those dealing with behavioural and psychosocial aspects of chronic pain management, was limited and GPs felt under pressure to do something whilst patients waited, usually increasing opioid therapy. Referrals allowed some alleviation of clinical responsibility but were made more in hope than in expectation.

*‘…every time I send somebody to chronic pain* [clinic] *they come out with more medication, or injections.’* [GP, female; Leeds; 319]

Patients could go through several cycles of referrals and new treatments, and of building up hopes and being disappointed, so that each cycle reinforced a sense of futility.

*‘Well all they do really is refer me to see a specialist, which was what they did in the first place, referred me to see a specialist and give me tablets… It’s like you are banging your head against the wall, you get fed up of going there… it’s a waste of my time, it’s a waste of their time because they’re not interested…’* [Male, 47 yrs, maintained on strong opioids, F1; 365–392]

Long term prescribing often continued because of a perceived lack of alternative options, with neither party feeling in control nor achieving satisfactory outcomes.

*Continuity of care*. Third, GPs were aware of the needs to get to grips with the key issues in patient narratives and to explore beliefs or behaviours which influenced their experience of pain. Some responded by trying to establish continuity through investing more time in patients with chronic pain.

*‘I think it’s good to take ownership…, because it’s that collusion of anonymity thing isn’t it? As soon as someone gets sort of uncomfortable they will shift to a different prescriber and they will push them along a certain course and they’ll go, I don’t like that, and they’ll go to a different one. And I honestly think it’s like a ship without a rudder and it’s just going round and round in circles.’* [GP, male, Leeds; 545]

Patients also highlighted problems with the lack of continuity and did not feel their needs could be properly addressed if their doctors were unfamiliar with their records. However, taking responsibility and trying to establish continuity had two potential drawbacks. It had implications for practice workload and might be perceived as unfair by colleagues because it reduced availability to see other patients:

*‘Would you feel guilty, you know, if you were bringing back a patient each week? And I think I’d have a bit of that because I think you don’t want to appear as if you’re seeing someone over and over again to your colleagues. I think that… certainly with me that I don’t want to be seen as that doctor that brings someone back every week…’* [GP, male, Bradford; 371]

Continuity could also result in stagnation, with losses of impetus and ideas for change, making it difficult for GPs or patients to question decisions:

*‘She’s [the GP] known me for years and years and she, she’s really nice and, but sometimes, in a way, knowing them can be worse, because you don’t tend to question their judgement, you know, because you don’t want to offend, if you get what I mean?’* [Female, 37 yrs, reducing strong opioids, B3:305]

One GP described her discomfort with consultations dealing with chronic pain and acknowledged that she felt better equipped to deal with different problems. Therefore continuity appeared more likely to be productive if GPs possessed interests and skills in supporting patients with chronic pain.

*Mutuality and trust in the relationship.* Finally, mutuality and trust between patients and GPs provided a platform for negotiating wider aspects of the relationship and considering more holistic approaches to pain management. However, establishing some level of trust was difficult when GPs had formed fixed, negative attitudes towards patients. GPs anticipated that consultations with certain patients were going to be problematic; this suggested that the problems resided as much within the characteristics of the patient as in the pain itself.

*‘I’ve got different responses to different patients I’ve realised just thinking about them. There’s one or two that my heart just goes, “oh dear, here we go…” because I know it’s going to be a tough consultation.’* [GP, female, Leeds; 794]

There could be significant resistance to suggestions of reducing or stopping opioid treatment:

*‘They’ve come in to get more medication and that’s their agenda. And you’re sitting there with this other agenda which is, you’re taking too many tablets and I’ve got to now try and address that with you. That is like a completely… they’re opposed completely so you know, they’ve come in with one thing and they know it’s going to be a battle, they’ve already got their hackles up.’* [GP, female, Bradford; 1010]

GPs often suspected they were faced with patients being managed for an incompletely formulated problem, especially in the presence of known or suspected mental health problems. Although they recognised the dangers of continuing or escalating opioid prescribing in such circumstances, GPs said that prescribing represented a means to establish trust so that non-pharmacological approaches could be considered then or at a later point.

*‘And then once you’ve got to that point and they’re still struggling with pain it’s almost having that conversation where you can then start to say, well maybe it’s not completely physical…’* [GP, male, Leeds; 343]

Some recognised this as a sub-optimal strategy as biopsychosocial approaches should ideally have been addressed at an earlier stage.

## Discussion

Patient needs for pain relief, an explanation of their symptoms and help to improve or maintain their quality of life were often unmet amongst those we interviewed. All GPs expressed dissatisfaction with some aspect of their approach to chronic pain management, be it with their own consultation skills, the limitations of the drugs they were prescribing, or the constraints of the clinical environment. The accounts in this study have highlighted problematic long-term prescribing, but we recognise that there will be many clinical encounters involving the non-problematic management of patients well controlled on a stable opioid prescription that is appropriate to their condition. We encountered a now-familiar story of dissatisfaction on both sides – patients experiencing repeated consultations that did not meet their needs, and GPs describing frustration with this state of affairs and yet with no clear idea of how to break out of it.

The resulting transaction was an impasse with the features of failure of satisfactory management of a long-term condition. The most striking manifestation of this failure was that both patients and clinicians recognised that they did not share a mutually-agreed plan of action, instead negotiating change through a series of short-term, often emotionally-charged consultations.

Three other themes in the clinical encounter confirm this formulation. First is an awareness of failure to resolve the tension between patient-centredness and evidence-based practice [37], generating uncertainty about responsibility for day-to-day symptom management [38]. Second is ambivalence about the place of relational continuity – valued in one way to maintain consistency but also seen as contributing to the nature of the impasse [39]. And finally there is an awareness of the lack of mutuality in the relationship – things not being understood, or if understood then not said: this is an important deficit given the centrality of trust to long-term care [40].

Given these circumstances, the continued prescription of an opioid can seem like the least-worst option for both parties, but becomes the main – if unintended – mechanism for preventing resolution. It creates an atmosphere of pseudo-mutuality and pseudo-control for both parties – the patient has got what they requested and the doctor has acted in role. The medication can reduce symptoms of distress and anxiety, at least transiently. And the act of giving and receiving the prescription can look like active engagement with the main dilemma but is in fact a form of avoidance, one of a number of strategies employed as means of not getting uncomfortable uncertainties and differences of opinion into the open [41].

### Strengths and limitations

We studied a sample of patients which may be atypical (more women; mainly prescribed strong opioids) although we did achieve some balance in trajectories. An alternative of asking GPs to select patients could have resulted in a different selection bias. There was likely under-representation of patients from black and minority ethnic groups. We also studied self-selected GPs who may have had greater interest in this issue, although our invitation specifically sought GPs with a range of interests and only a minority in our sample self-declared interests in pain. We studied GPs and patients in one geographical area within the UK National Health Service; however, similar concerns about prescribing decisions and actions are emerging elsewhere in other healthcare systems [42]. Our understanding of prescribing trajectories was based upon subjective accounts of diagnoses and opioid prescription strength and duration, with discrepancies between prescription records and what patients reported taking, although this is common for many prescribed medications.

### Comparisons with existing literature

Our patient accounts are broadly consistent with those in a recent meta-ethnography of qualitative studies, particularly in the ‘adversarial struggle’ patients faced in constructing explanations for their symptoms and negotiating the healthcare system [30]. Similarly, GPs would recognise such experiences and have further concerns around adverse effects, addiction, misuse and probity of prescribing [21, 43]. Variations in reported practice have been linked to training in pain management and experience, including adverse events [31].

Esquibel and Borkan also compared the experiences of primary care physicians and patients in one US Family Care Center, separately interviewing each in 21 dyads [44]. There were many similarities to our findings, such as challenges in legitimising non-objective pain and physician feelings of inadequacy as care providers in treating pain. However, the problems we uncovered with the lack of management plans and discontinuity were less apparent, probably because all patients held contracts for opioid treatment of chronic pain and the physicians knew and nominated each patient interviewed.

### Implications for research and practice

Our findings suggest that GPs would be receptive to guidance on and support with the management of chronic, non-cancer pain and opioid prescribing. The content of educational interventions should draw upon existing good practice guidance [45], and preventive strategies suggested by our study. These include: early recognition of at-risk patients who might benefit from more anticipatory and structured management; checking patient expectations of opioid therapy and advising on their limited benefit in chronic pain [46]; and reconsidering strategies of prescribing opioids to establish false therapeutic relationships.

For those already prescribed opioids, our findings indicate the need for a systematic approach to each patient that is not negotiated ‘on the hoof’ during individual consultations. A first step in primary care is practice-level reviewing of prescribing and potential non-pharmacological approaches to chronic pain management; a plan can then be formulated that is not over-influenced by the short-term consultation-by-consultation approach. Consistency in delivering this plan requires clear communication, including agreement of a shared aim within the general practice team and informational continuity for individual patients. At the same time, it cannot be assumed that relational continuity will work in the patient’s best interests, given the risks of perpetuation of ineffective clinical strategies [47]. Therefore, practices should consider matching patients with more problematic issues to GPs with greater skills in pain management. Subsequent consultations require open and explicit discussions of the element of management plans that is doctor-driven, including the possibility of non-negotiated reduction or stopping of opioids, to establish the boundaries of clinical responsibility for prescribing and reduce some of the mistrust due to lack of openness about plans.

General practice strategies to prevent or manage problematic opioid prescribing also need support from commissioned specialist services which should include non-drug interventions. There are transferable elements of collaborative care approaches, effective for other long term conditions, which enable active communication between GPs and specialists to monitor and plan patient care [48]. However, there is a need for rigorously developed and evaluated interventions to change GPs’ chronic pain management and prescribing behaviours.

## Conclusions

Problematic prescribing occurs when patients experience repeated consultations that do not meet their needs and GPs feel unable to negotiate alternative approaches to treatment. Therapeutic short-termism is perpetuated by inconsistent clinical encounters and the absence of mutually-agreed formulations of underlying problems and plans of action. Apart from commissioning improved access to appropriate specialist services, general practices should also consider how they manage problematic opioid prescribing and be prepared to set boundaries with patients.

## Appendix 1: Patient interview topic guide

Introduction to the interview

Statement about the study aims: purpose is to help us to understand how, over time, your experiences of being prescribed painkillers have changed. We are interested to know how you came to be on painkillers in the first place and what has happened since then; What I know about you already (from the opt-in form and why you have been selected to take part); format; length; breaks; section of the information leaflet that talks about what will happen if anything discussed is deemed as a risk.

1. The illness/prescribing career
  - List all medications, doses and conditions
  - Could you put me in the picture, in a few sentences, telling me about when your pain began?
    *Prompts: earliest memory of being in pain; when became aware of it; how often was it happening and for how long?*

- Could you talk me through, from the beginning, what medication you have been prescribed to help you with the pain that you get?

*Prompts: At the beginning (what happened and why); how prescription has changed and what led up to these changes (key events/triggers); who has been involved in decisions about these changes, why; how feel about your prescription ‘path; why did you get to where you are (rather than how)*

2. Consultation dynamics
  - Can you tell me how you feel about the way in which your GP/general practice has managed your pain?

*Prompts: how arrived at diagnosis (i.e. investigations), how do you feel about this (diagnosis and route taken); who has been involved in managing your pain (e.g. which health professionals, when in the prescribing career); how involved do you feel in decisions that influence how your pain is managed; how do you feel about taking painkillers; have there been any attempts to change (decrease, stop or increase) your painkillers, who by and why, how do you feel about this; who makes decisions about changes; more about emotional response to changes (recent step-ups/downs alterations in dosage) e.g. disapproval in consultation, responsibility and attribution;*

3. Hopes/expectations of prescription
  - Can you tell me what impact your painkiller prescription has on your daily life?
    *Prompts: How do tablets help you to do the things that you want to do; what you do, what you can do and what you would like to do (quality of life/employment/social); do they affect your mood, in what way, how feel about this; other physical effects and/or unwanted effects, how feel about this; how important is your medication regime in your daily life, why, what would happen if you did not take them; to what extent do painkillers give you relief from pain, is this acceptable to you, will this change in the future, why, in what way, how do you feel about this; how do you feel about any unresolved pain, will this be different in the future, in what way, why, how do you feel about this*
  - Can you tell me what else you have tried to help manage your pain, besides painkillers?
    *Prompts: what, why, when in prescribing career, impact, how feel about this; importance of [aids] in relation to medication and why; what else will you do to manage your pain in future, why, how do you feel about this*
    Ending the interview
    Checks; prompts; what next

## Appendix 2: Patient scenarios for use in focus groups

Mr W: Current analgesia reported by patient: Fentanyl transdermal patch (100mcg) change every 2–3 days; tramadol 50 mg approx. 15 per day (750 mg daily – prescribed 400 mg); paracetamol prn

Mr W is a 40 year old married man with 3 teenage children. He currently works full-time in a semi-professional job. His low back pain started around 15 years ago. A scan led to a diagnosis of degenerative discs. A recent second scan shows worsening degeneration. He has no other current health problems except for low mood within the last 2 years.

He has been on numerous strengths and doses of painkillers since diagnosis (he does not get on well with anti-inflammatories). He has been prescribed strong opioids (Fentanyl patch) for the past 6 years, increased gradually over the past year. He has gradually increased the dose of tramadol himself, especially as he is ‘not getting the same relief that he used to get from his patch’. His wife is set against any further increases in opioids and says that his GP is letting her husband ‘self-prescribe’. Mr W complains that the increase in pain is limiting his ability to work, which is ‘what he lives for.’

Mrs R: Current analgesia reported by patient: co-codamol 30/500 mg x 2 qds; naproxen 250 mg tds; paracetamol prn

Mrs R is a 72 year widow who lives with her daughter and son-in-law. She has a long-standing history of osteoarthiritis. She had both hips replaced around 5 years ago. She suffered an MI last year, after which she moved in with her daughter. She has recently complained of an increase in the amount of pain she gets from her shoulder and knee joints. She feels that her GP has not listened to her when she asked for help with this in the past. She was recently referred to and completed physiotherapy classes. She says that these were ‘a waste of time.’ It was a massive effort for her to get there and the classes were often postponed at the last minute due to staff shortages. Around the same time, she was prescribed a low dose of tramadol but she says she has stopped taking it as it makes her ‘feel weird.’
